# Extended spectrum beta-lactamase (ESBL) strains of E. coli as a cause of urinary tract infections in hospitalized patients

**DOI:** 10.1186/2047-2994-4-S1-P121

**Published:** 2015-06-16

**Authors:** V Gerasimovska, B Gerasimovska-Kitanovska

**Affiliations:** 1 Vascular Acess, University Clinic of Nephrology, Skopje, The Former Yugoslav Republic Of Macedonia; 2 University Clinic of Nephrology, The Former Yugoslav Republic Of Macedonia

## Introduction

Urinary tract infections are the second most frequent infections of any organ and among the most common infections in adults. They may be community-acquired or nosocomial, mainly caused by Gram negative bacteria, the most frequent finding being E. coli.

## Objectives

The aim of our study was to determine the characteristics of urinary tract infections in hospitalized patients in the University Clinic of nephrology in Skopje, caused by ESBL positive E. coli.

## Methods

Medical records were reviewed retrospectively and demographic, laboratory and clinical data were obtained in patients with ESBL positive E. coli. Study included 80 patients, 40 with ESBL positive and 40 with ESBL negative strains of E. coli.

## Results

The groups did not differ in number, gender and age, but ESBL positive group was of older age and had more cases with urosepsis than the ESBL-negative group. Regarding the comorbidities, most of the patients had diabetes mellitus type 2- in 16 patients (40%) and 15 of them (37%) had chronic kidney disease. In most of the patients, antibiotics were used empirically before urine culture was obtained, which accounts for the high number of cephalosporins and hinolones used – 9 patients (22%), and amikacyn in 11 (27%), imipenem 5 (8%). Univariate analysis of risk factors associated with ESBL+ infections, identified diabetes mellitus, sepsis and previous hospitalizations are predictors of ESBL positive infections. When the three variables were entered into a multivariate logistic regression, diabetes and sepsis were found to be predictors of ESBL positive patients.

## Conclusion

Our results showed that ESBL-positive urinary tract infections were more frequently associated with sepsis, and that the index of comorbidities, although rather high in both groups, was not associated exclusively with ESBL positive strains.

It is important to provide prompt diagnosis in cases with ESBL positive urinary tract infections, because improper treatment is a factor for higher mortality.

## Disclosure of interest

None declared.

